# Sewage outburst triggers *Trichodesmium* bloom and enhance N_2_ fixation rates

**DOI:** 10.1038/s41598-017-04622-8

**Published:** 2017-06-29

**Authors:** Eyal Rahav, Edo Bar-Zeev

**Affiliations:** 1 National Institute of Oceanography, Israel Oceanographic and Limnological Research, Haifa, 31080 Israel; 2 The Jacob Blaustein Institutes for Desert Research, Zuckerberg Institute for Water Research (ZIWR), Ben-Gurion University of the Negev, Sede Boqer Campus, Beer Sheva, 84990 Israel

## Abstract

The southeastern Mediterranean Sea (SEMS) is a warm and sunlit marine environment with low ambient N concentration, thus considered ideal for diazotrophy by autotrophic diazotrophs such as *Trichodesmium*. Despite the favorable conditions, N_2_ fixation rates are often low and *Trichodesmium* has hardly been spotted in the SEMS. This study reports on the occurrence of a *Trichodesmium* bloom in the SEMS which was ascribed to *T. erythraeum* according to DNA fingerprinting of the *nifH* gene. We found that this bloom (1407 ± 983 cells L^−1^) was triggered by an intense outburst of raw sewage that supplied high concentrations of N, P and dissolved organic carbon (DOC), which resulted in low N:P (~12:1) and exceptionally high C:P (~1340:1) ratios. We surmise that these conditions provided favorable conditions for *Trichodesmium* bloom to form via mixotrophic metabolism. As a result, a fourfold increase in N_2_ fixation was recorded, which contributed ~70% to new primary production and spur a sharp increase in phytoplankton activity and biomass. The conclusions of this study point on a new paradigm for bloom-forming *T. erythraeum* which is tightly linked to anthropogenic sources and prompt microbial productivity in oligotrophic marine environments such as the SEMS.

## Introduction

Coastal environments are routinely exposed to runoffs from anthropogenic sources, including municipal, industrial and agricultural waste^1, 2^. These abrupt runoffs are often untreated and contain pathogens, organic and inorganic nutrients, heavy metals and detergents and are thus considered major threats to marine ecosystems^3, 4^. Such discharges were previously shown to alter food-web dynamics in coastal environments^1, 5, 6^. For example, sewage outbursts introduce nutrients that can stimulate bottom-up effects^7^ and shift the phytoplankton community structure to form large blooms of toxic dinoflagellates or other harmful algal species^8–10^.

The surface water of the southeastern Mediterranean Sea (SEMS) is a warm, sunlit marine environment with ultra-oligotrophic conditions, resulting in low bacterial and phytoplankton biomass and low productivity^11–13^. Phytoplankton productivity is considered either N-limited^14^ or N and P co-limited^15, 16^, whereas bacteria are either P-limited^17^ or C-limited^14^. These conditions are supposedly ideal for diazotrophy to occur by autotrophic diazotrophs that can fix C and N using the sunlight energy^18^. Although the diazotrophic community in the eastern Mediterranean Sea is highly diverse^14, 19, 20^, typical N_2_ fixation rates are constantly low (<0.4 nmol N L^−1^ d^−1^)^13, 14, 21–23^, whereas large scale *Trichodesmium* spp. were only reported once in the northeastern Mediterranean Sea^24^ and recently in the Gulf of Gabes (Tunisia)^25^.

*Trichodesmium* spp. is considered to be one of the most dominant nitrogen-fixing microorganisms in marine systems^26, 27^. It was previously reported that large *Trichodesmium* blooms may fix CO_2_ and N_2_ at high rates reaching 640 pg C cell^−1^ d^−1^ and 29 pg N cell^−1^ d^−1^, respectively, thereby prompt the microbial food web^28^. The formation of *Trichodesmium* blooms are often hindered by the availability of trace metals (mainly Fe) and other nutrients (mainly P)^29, 30^, water temperature^31^, light availability^32, 33^, high O_2_ levels^34^, as well as other physiochemical water characteristics^35^. However, the conditions restricting the formation of *Trichodesmium* spp. blooms in the SEMS, which is potentially ideal for diazotrophy, are currently unknown^18^.

In this study, we focused on the possible links between sewage outburst and the development of *Trichodesmium* bloom and corresponding microbial community structure. To this end, we followed a *Trichodesmium erythraeum* bloom in the coastal water of the SEMS which was triggered by an outburst of municipal sewage. During this event, we followed the temporal dynamics of *T. erythraeum* and N_2_ fixation, as well as picophytoplankton, diatoms, dinoflagellates, and heterotrophic bacteria. Our results point on the possible impacts of sewage outbursts that flow into ultra-oligotrophic marine environments such as the SEMS.

## Material and Methods

### Study site and sample collection

Seawater samples (~20 L) were collected from the surface water (~0.5 m) of the southeastern Mediterranean coastline (32°49′34 N, 34°57′20E) during wintertime. Up to ten measurements were carried out; (i) two-three prior the sewage outburst, (ii) five during the event, and (iii) two immediately after the sewage outburst had ended. Seawater was sampled for inorganic nutrients, dissolved organic carbon, chlorophyll-*a* (as an algae proxy), picophytoplankton abundance including *Synechococcus* + *Prochlorococcus* (collectively referred to as autotrophic cyanobacteria) and picoeukaryotes, heterotrophic bacterial abundance and microphytoplankton abundance (diatoms and dinoflagellates). Furthermore, water samples were collected for *Trichodesmium* spp. abundance, diazotrophic diversity (*nifH* gene analysis), primary production, bacterial production and N_2_ fixation measurements.

### Inorganic nutrients and dissolved organic carbon (DOC)

Water samples for NO_2_ + NO_3_, PO_4_ and Si(OH)_4_ concentrations were collected in 15 mL acid-washed plastic scintillation vials and placed immediately in a −20 °C freezer until analysis. Inorganic nutrient values were determined using the segmented flow Seal Analytical AA-3 system^36^.

Samples for DOC concentrations (40 mL) were collected in septum-cap glass vials and were acidified with concentrated (32%) hydrochloric acid (HCl) at a ratio of 1:1000 and stored in the dark at 4 °C until analysis. Samples were analyzed on a Shimadzu TOCV analyzer with a precision of 2 μmol L^−1^.

### Chlorophyll-a (Chl-a)

Seawater samples (300 mL) were filtered through a Whatman GF/F filter (~0.7 μm nominal pore size) and kept at −20 °C in the dark. Filters were extracted overnight in acetone (90%) and determined by the non-acidification method^37^ using a Turner Designs (Trilogy) fluorometer with 436-nm excitation and 680-nm emission filters.

### Picophytoplankton abundance

Water samples (1.7 mL) were fixed with 50% glutaraldehyde (Sigma-Aldrich G7651), snap-frozen in liquid nitrogen and stored at −80 °C. Picophytoplankton abundance was determined based on the orange auto-florescence of phycoerythrin and the red auto-florescence of chl-*a* ^38^. Heterotrophic bacterial abundance was measured by staining the sample with 1 μl of SYBR green (Applied Biosystems cat #S32717) followed by a 10-min incubation in the dark. All samples were analyzed by an Attune® Acoustic Focusing Flow Cytometer (Applied Biosystems) equipped with 488-nm and 405-nm lasers. For the size standard, 1-μm beads (Polysciences) were used.

### *Trichodesmium*, diatom and dinoflagellate abundance

Cell densities were determined after concentrating 1–5 L of surface seawater and counting three subsamples. Cell numbers were estimated using a Sedgewick-Rafter Cell (S50) and an epi-fluorescence light microscope (Olympus BH-2) using 20–40X magnification.

### Extraction and sequencing of the *nifH* gene

Samples (1–5 L) were filtered through 0.2-μm Supor filters (PALL Corp.) and placed in a sterile DNase/Rnase Free Whirl-Pak bag. The samples were snap-frozen in liquid nitrogen and stored at −80 °C. DNA was extracted using the phenol-chloroform method according to Man-Aharonovich^19^. Nitrogenase Fe protein transcripts (*nifH*) were amplified using a nested PCR strategy^39^. A paired-end sequencing of DNA was performed on an Illumina MiSeq platform at the Research and Testing Laboratories (Lubbock, TX, USA).

### Sequencing analysis

Merged Illumina reads were quality filtered and analyzed with the Quantitative Insights Into Microbial Ecology (QIIME) pipeline^40^. The remaining reads were binned into operational taxonomic units (OTUs) and defined at 97% similarity using the UCLUST algorithm^41^. Taxonomy was assigned with BLAST and a database of *nifH* sequences from Heller *et al*.^42^. Phylogenetic trees were generated with FastTree in QIIME^43^ and visualized with the Interactive Tree of Life (IToL) and Topiary Explorer v1.0 packages.

### Primary production (PP)

Photosynthetic carbon fixation rates were estimated using ^14^C incorporation^44^. Water samples were analyzed in triplicates with dark and zero-time controls. Samples (50 mL) collected at each time point were added to polycarbonate bottles (Nalgene) containing 5 μCi of NaH^14^CO_3_ (Perkin Elmer, specific activity 56 mCi mmol^−1^) and incubated for 4 h under ambient natural illumination and temperature. To determine the quantity of the added radioactivity, 50 μl of each sample was immediately mixed with 50 μl of ethanolamine and stored for analysis. The incubations were terminated by filtering the spiked seawater through GF/F filters (~0.7 μm nominal pore size) at low pressure (~50 mmHg). The filters were incubated overnight in 5-mL scintillation vials containing 50 μl of 32% HCl in order to remove excess ^14^C-bicarbonate. After adding 5 mL of scintillation cocktail (Ultima-Gold) to each vial, the radioactivity was measured using a TRI-CARB 2100 TR (Packard) liquid scintillation counter.

### Bacterial production (BP)

Rates were estimated using the [4,5-^3^H]-leucine incorporation method^45^. Three aliquots (1.7 mL each) from each water sample were incubated with 100 nmol of leucine L^−1^ (Amersham, specific activity 160 Ci mmol^−1^) for 4 h at ambient temperature in the dark. Samples treated with trichloroacetic acid (TCA) were used as a control. The incubations were terminated with 100 μL of TCA (100%), followed by micro-centrifugation. After adding 1 mL of scintillation cocktail (Ultima-Gold) to each vial, the samples were counted using a TRI-CARB 2100 TR (Packard) liquid scintillation counter. A conversion factor of 3 kg C per mole leucine incorporated was used, assuming an isotopic dilution of 2.0^46^.

### Dinitrogen (N_2_) fixation

Rates were measured in triplicates using the ^15^N_2_-enriched seawater protocol^47^. ^15^N_2_-enriched seawater was prepared by injecting 1:100 (vol:vol) ^15^N_2_ gas (99%) into degassed (MiniModule G543) and filtered (Polycarbonate 0.2 μm) seawater collected at the study site. The enriched seawater stock was shaken vigorously in order to completely dissolve the ^15^N_2_ gas, and aliquots (225 mL) were then added to triplicate experimental Nalgene bottles (4.6 L). Following 24 h of incubations under ambient light and temperature conditions, the samples were filtered through pre-combusted (450 °C, 4.5 h) GF/F filters and dried in an oven at 60 °C overnight. The samples were then analyzed using a CE Instruments NC2500 elemental analyzer interfaced to a Thermo-Finningan Delta Plus XP isotope ratio mass spectrometer (IRMS). For isotope ratio mass spectrometry, a standard curve to determine N mass was performed with each sample run.

### Statistical analyses

Data are displayed as averages; error bars signify one standard deviation (n = 3–5). The relationships between the different environmental and physiological variables were determined with a Pearson correlation test (P < 0.05). All tests were performed using the XLSTAT software.

## Results and Discussion

**Figure 1:**
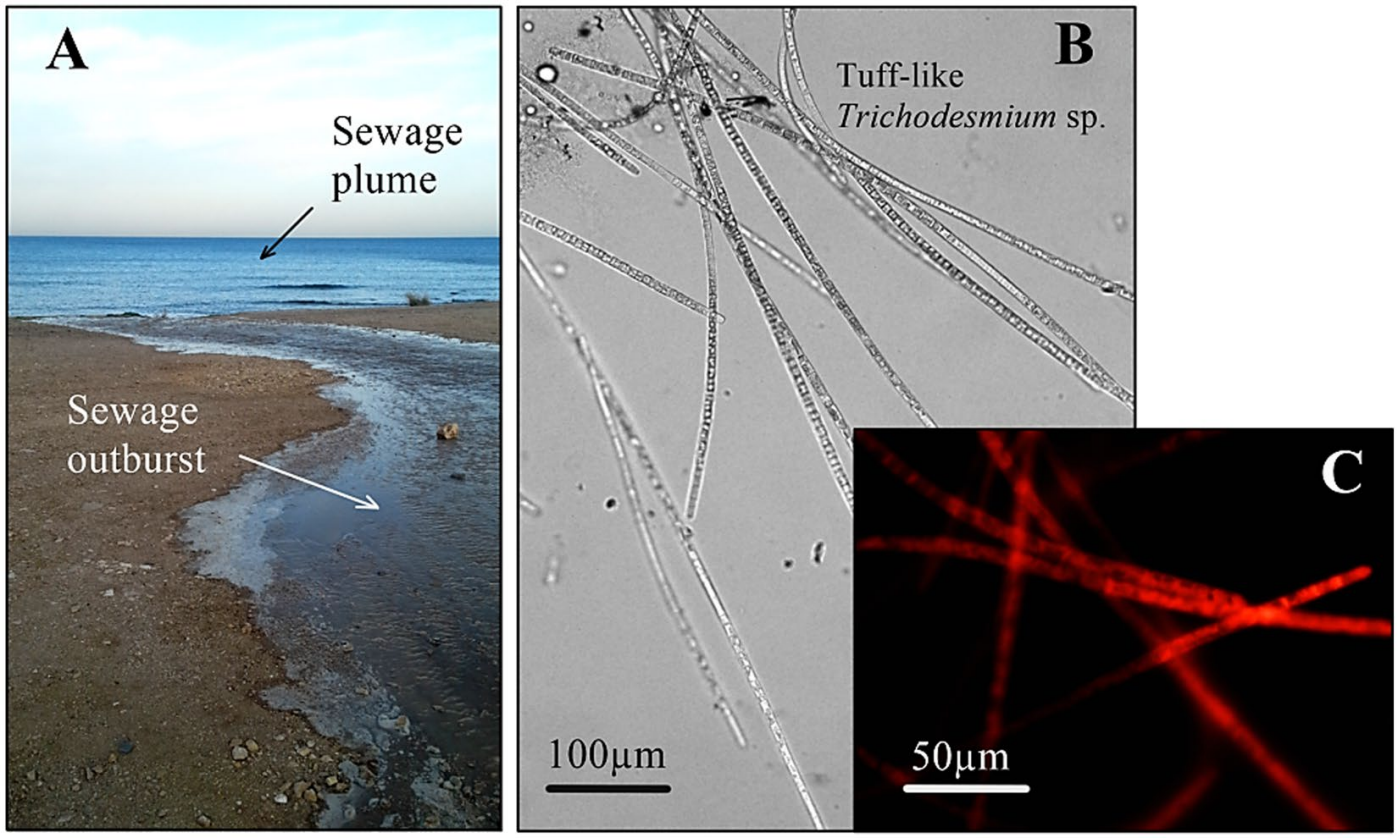
(**A**) Sewage outburst into the coastal SEMS water in February 2015; (**B**) dense *Trichodesmium* spp. colonies observed under bright field microscopy; (**C**) *Trichodesmium* spp. autoflorescence following excitation with a phycocyanin filter, in which gas vacuoles are seen as black voids. The picture was taken by E. Rahav.

**Figure 2:**
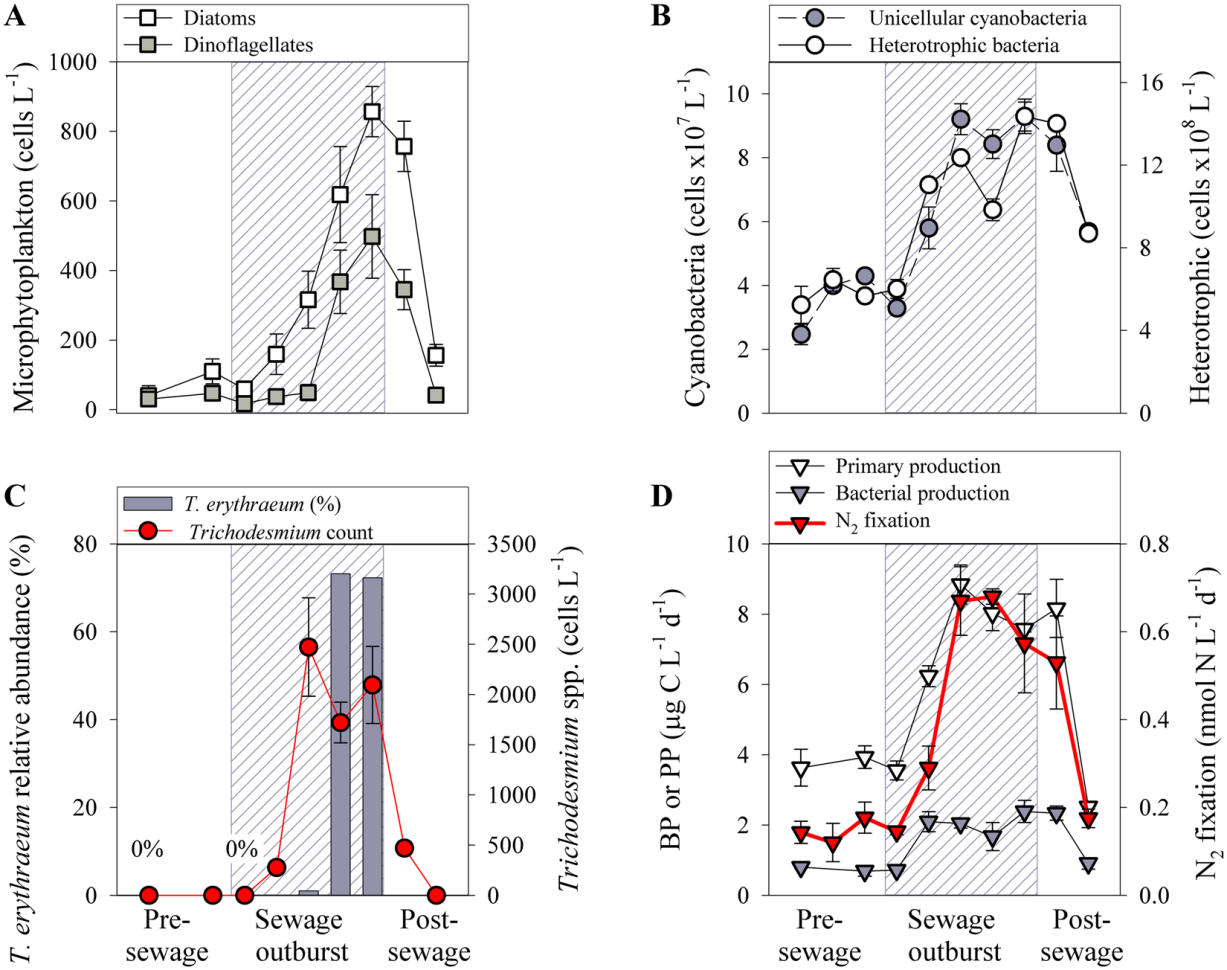
The temporal distribution of (**A**) microphytoplankton (**B**) autotrophic and heterotrophic bacteria, (**C**) the relative abundance of *T. erythraeum* (from all *nifH* OTUs) and corresponding microscopical counts, (**D**) bacterial and primary production (BP and PP, respectively) and N_2_ fixation rates. Measurements were taken prior to, during, and after a sewage outburst event at the coastal SEMS during wintertime (January to March 2015). Values presented are averages and their corresponding standard deviations (n = 3–5).

Untreated sewage outbursts into the SEMS coastal environment occur a few times every winter, usually due to deficiencies in the sewage network and/or overloading of the sewage drainage system by storm-water runoffs (Fig. 1A). The introduction of high loads of organic matter and nutrients into oligotrophic coastal environments such as the SEMS may often result in the formation of phytoplankton blooms (Figs 1B,C and 2). The formation of these blooms may alter microbial food-web dynamics^48^ and release different harmful toxins^49^, as well as hinder the performance of large-scale seawater desalination and power plants^50^.

### Environmental conditions of the study site during wintertime

The coastal water prior to the outburst event exhibited typical SEMS wintertime characteristics (Table 1), with cold (18.3 ± 0.7 °C), windswept (10 ± 3 knots) and well-oxidized (190 ± 10 μM) water. Inorganic nutrient levels, namely NO_2_ + NO_3_, PO_4_ and Si(OH)_4_), were low (close to detection limit for P and up to ~2 μM) as well as dissolved organic carbon (DOC, 10 ± 5 μM). These physicochemical conditions resulted in low phytoplankton biomass (0.32 ± 0.05 μg chl-*a* L^−1^), which is typical for this region^14, 23^. *Synechococcus* were the most abundant phototroph (3.9 ± 1.2 cells × 10^7^ L^−1^), whereas diatom (116 ± 43 cells L^−1^, mostly *Chaetoceros* spp.) and dinoflagellate (34 ± 13 cells L^−1^, mostly *Ceratium* spp.) abundances were 5–6 orders of magnitude lower (Fig. 2A,B). At this time, *Trichodesmium* spp. could not be detected (Fig. 2C). The low phototrophic abundance resulted in scant PP rates (3.3 ± 0.6 μg C L^−1^ d^−1^, Fig. 2D). Heterotrophic bacterial abundance was higher by ~tenfold relative to *Synechococcus* (64 ± 14 cells × 10^7^ L^−1^, Fig. 2B). However, BP rates were low (0.8 ± 0.2 μg C L^−1^ d^−1^) and constituted ~25% of the PP (Fig. 2D). N_2_ fixation rates were also low (0.15 ± 0.02 nmol N L^−1^ d^−1^, Fig. 2D) and similar to previously reported values from this study area^14, 23^. We attribute the low N_2_ fixation rates to alpha, gamma and delta—proteobacteria diazotrophs as recently reported for the coastal SEMS^14^.

**Table 1:** The physiochemical and biological characteristics of the SEMS coastal water during a sewage outburst event and during typical wintertime (ambient) conditions.

Variable tested	Units	Sewage outburst	Ambient conditions	Sewage: Ambient (ratio)
Temperature	°C	18.1–20.6	17.3–19.2	1.1
Wind	Knots	0–4	7–13	0.2
Salinity	–	39.0–39.4	39.0–39.6	1.0
NO_2_ + NO_3_	μM	2.11–2.50	0.64–1.37	2.1
PO_4_	μM	0.14–0.29	0.02–0.07	4.2
Si(OH)_4_	μM	0.41–2.48	1.12–1.94	0.9
N:P	Ratio	8.6–15.1	19.6–32.0	0.5
DOC	μM	188–339	6–13	25
O_2_	μM	141–160	177–202	0.8
Chl-*a*	μg L^−1^	0.45–0.59	0.23–0.36	1.6

### Coastal conditions in the SEMS during a sewage outburst event

During February 2015, raw and untreated domestic sewage was continuously and intensely (thousands of L h^−1^) discharged for five days into the coastal water of the SEMS due to various impairments in the municipal drainage system of Haifa, Israel (Fig. 1A). During this period, water temperature and salinity were not affected and remained typical to the season, and only a weak breeze (<4 knots) was recorded throughout the sewage outburst event (Table 1). Concurrently, NO_2_ + NO_3_ (2.3 ± 0.2 μM) and PO_4_ (0.2 ± 0.1 μM) levels increased by twofold and fourfold respectively (Table 1). This change in NO_2_ + NO_3_ and PO_4_ resulted in a reduced N:P ratio (~12), which is lower than the colonial 16:1 Redfield ratio. DOC levels have also increased by 25-fold relative to the ambient pre-sewage conditions (268 ± 56 μM).

Following the sewage outburst, a large tuff-like *Trichodesmium* spp. bloom developed, reaching densities of 1407 ± 983 trichomes L^−1^ (Figs 1B and 2C). Genetic fingerprinting of *nifH* indicated that the most abundant diazotrophic specie was *Trichodesmium erythraeum* (*T. erythraeum*), constituting >70% of all the diazotrophic OTUs in the sewage plume (Fig. 2C). Concurrently, the relative OTUs of other diazotrophs, such as heterotrophic proteobacteria (i.e., *Desulfobacter sp*.) and cyanobacteria (i.e., *Leptolyngbya sp*.), were significantly lower (<1%). Our measured *T. erythraeum* densities were in agreement with those previously reported in the northeastern Mediterranean Sea (Aegean Sea) in September 2010^24^ and from a three-year survey in the Gulf of Gabes (Tunisia)^25^. However, these cell numbers were below the densities reported for large-scale *Trichodesmium* spp. blooms in other marine environments that ranged from 3000 to >10000 trichomes L^−1^ ^51–53^.

Together with the sharp and significant rise in *T. erythraeum*, N_2_ fixation rates increased by ~fourfold (Fig. 2D). Therefore, we attributed the lion’s share of the corresponding N_2_ fixation rates to *T. erythraeum* (Table 2). This relationship yielded specific N_2_ fixation rates per trichome of ~3.5 nmol N trichome^−1^ d^−1^, which are at the lower end of the values reported from other oceanic systems where *Trichodesmium* spp. prevails^53–55^, or from *T. erythraeum* monocultures^56^. Still, it should be noted that not all cells within a *Trichodesmium* filament take an active role in N_2_ fixation^34^. Therefore, it is possible that the *T. erythraeum* filaments observed here had fewer N_2_-fixing cells (out of all vegetative cells) than in other marine systems, which would result in lower specific N_2_ fixation rates compared with other marine systems.

**Table 2:** Pearson linear correlation matrix between the different variables tested before, during, and after the *Trichodesmium* bloom in the SEMS from January to March 2015.

	N_2_ fix	N:P	DOC	Chl-*a*	BA	BP	PP	*Syn*.	Diat.	Dino.	*Tricho*.
N_2_ fix	**1**										
N:P	−0.67	**1**									
DOC	**0.94**	**0.76**	**1**								
Chl-*a*	**0.92**	−**0.87**	0.67	**1**							
BA	**0.56**	−0.41	**0.83**	**0.56**	**1**						
BP	**0.76**	−**0.76**	**0.91**	**0.74**	**0.69**	**1**					
PP	**0.94**	−**0.84**	0.46	**0.96**	0.51	**0.87**	**1**				
*Syn*.	**0.92**	−0.57	**0.51**	**0.77**	**0.66**	**0.82**	**0.86**	**1**			
Diat.	**0.76**	−0.37	0.58	0.62	**0.68**	**0.73**	**0.71**	**0.78**	**1**		
Dino.	0.67	−0.30	0.44	0.55	0.35	0.62	0.59	0.68	**0.97**	**1**	
*Tricho*.	**0.89**	−**0.59**	**0.92**	**0.79**	0.47	0.62	**0.80**	**0.83**	0.58	0.52	**1**

Values in bold show statistically significant relationships between the variables (alpha = 0.05, n = 9–10). *Syn*.: *Synechococcus*; Diat.: diatoms; Dino.: dinoflagellates; *Tricho*.: *Trichodesmium*.

Concomitant with the sewage outburst event and the *T. erythraeum* bloom, chl-*a* levels positively and linearly increased by 60% (0.51 ± 0.06 μg L^−1^) and PP by 230% (7.77 ± 0.97 μg C L^−1^ d^−1^) compared with the ambient concentrations (Tables 1 and 2), indicating a significant increase in phototrophic biomass. Specifically, these changes were even more evident in the increased abundances of diatoms (~fourfold, mostly *Asterionellopsis glacialis* and *Leptocylindrus danicus*) and dinoflagellates (~sevenfold, mostly *Ceratium* spp.) following the development of the *T. erythraeum* bloom (Fig. 2A–C, Table 2). In contrast to the significant rise in microphytoplankton abundance, heterotrophic bacteria only increased by 1.9-fold (Fig. 2B); however, BP rates increased by threefold relative to pre-discharge production rates. This decoupling between the moderate rise in total heterotrophic bacteria and the significant increase in BP rates, along with the high DOC levels that were introduced with the sewage (Table 1), suggests that some phototrophs might also utilize these organic substrates by switching to mixotrophic nutrition^57, 58^.

### The importance of *Trichodesmium* to new production and microbial biomass in the SEMS

*Trichodesmium* blooms are known to release bioavailable nitrogen into the surrounding water, leading to enhanced growth of non-diazotrophic cyanobacteria and microphytoplankton^22^. Indeed, the appearance of *T. erythraeum* in the SEMS water was positively and significantly coupled with phototrophic biomass (as chl-*a*), *Synechococcus* abundance, PP and N_2_ fixation (Table 2). It was also positively correlated with microphytoplankton abundance, although not significantly (Table 2). We estimated that the contribution of *T. erythraeum* to PP via N_2_ fixation (based on C:N Redfield stoichiometry) reached 68 ± 27%. This contribution is ~sevenfold higher than the percentage usually reported in the same study area and season during non-bloom conditions (~10%)^23^, and 30–140-fold higher than that reported from the offshore waters of the eastern Mediterranean Sea (~0.5–2%)^13, 20, 23, 59, 60^. We suggest that the new N derived from *T. erythraeum* N_2_ fixation, along with the additional nutrients that were introduced by the sewage, may induce primary and bacterial production rates, resulting in prompted microphytoplankton growth (Fig. 2, Table 2). Diatoms and dinoflagellates abundance markedly increased at day 4 of the sewage outburst and 1-2 days since the appearance of *Trichodesmium* (Fig. 2). Therefore, we surmised that it is likely that microphytoplankton growth was linked to *Trichodesmium* and the corresponding elevated N_2_ fixation rates. Similarly, a *Trichodesmium* bloom in the near-shore waters of Goa (western India) led to *Asterionella japonica* and *Nitzschia closterium* blooms^61^. In the Gulf of Mexico, *Trichodesmium* spp. triggered a bloom of the toxic dinoflagellate *Karenia brevis* ^62^. Additionally, recent nanoscale secondary ion mass spectrometry (NanoSIMS) analyses showed that small-size autotrophic picophytoplankton accumulated ^15^N-derived *Trichodesmium* spp. in the waters of the southwestern Pacific Ocean around New Caledonia^22^. Furthermore, large-scale *Trichodesmium* blooms were reported to swiftly diminish via grazing by harpacticoid copepods^63^, by viral lysis^64^ or by a genetically controlled programmed cell death^28, 53, 65^. These swift bloom demise (~50% of biomass loss within 24 h)^28^ may fuel higher trophic levels and significantly change the microbial community structure.

### Factors controlling the activity and bloom formation of *Trichodesmium* in the SEMS

*Trichodesmium* growth and activity may be affected by several physiochemical characteristics and currently remain a matter of debate in many oceanic regions^27, 52^. Environmental factors, such as sea surface temperature, *p*CO_2_, irradiance, a quiescent sea state and the availability of nutrients such as P and Fe, have all been suggested as controlling factors for *Trichodesmium* blooms^29, 30, 33, 66–70^. Currently, the reasons why *Trichodesmium* blooms do not develop in the SEMS, despite being a potentially ideal marine environment with beneficial characteristics for diazotrophy, are still unknown. Our findings suggest that apart from the calm sea, sunlit conditions and the somewhat low N:P ratio (12:1), two mechanisms may have been in play and triggered the development of the *T. erythraeum* bloom: (i) The high concentrations of N and P (at a low N:P ratio) that were introduced to the coastal environment with the sewage supplied the initial cellular metabolic needs for the growth and proliferation of *T. erythraeum* cells. (ii) The high DOC concentration that was supplied with the sewage may have provided assimilable carbon source to the coastal environment. A recent study showed that *Trichodesmium*, as other unicellular diazotrophs^71, 72^, can utilize DOC as an available carbon source^73^ and therefore may provide an alternative energy source to fix N_2_ and form blooms. Assuming that *T. erythraeum* cells are mixotrophs^73^, we suggest that *Trichodesmium* blooms could be stimulated by these types of sewage outbursts in the oligotrophic SEMS. Furthermore, during the bloom, the high concentrations of DOC resulted in an exceptionally high C:N:P ratio (~1340:12:1) relative to the measured ambient ratio during the non-bloom period (~233:25:1). We suggest that these conditions may have induced N-limiting yet C-rich conditions that prioritized mixotrophic metabolism by *Trichodesmium*. It should be noted that while Benavides *et al*.^73^, suggested that natural *Trichodesmium* populations may use alternative mixotrophic nutrition and assimilate DOC as a primary C source, such an addition will not necessarily trigger a *Trichodesmium* bloom in the SEMS. In fact, a recent study from the coastal SEMS reported that heterotrophic diazotrophy was stimulated by a DOC + N + P addition, whereas *Trichodesmium* spp. was not observed^14^. We propose that additional materials such as trace metals and vitamins were introduced with the sewage outburst and played mutual roles in the formation of the *T. erythraeum* bloom that was reported here. Still, the reasons as to why *Trichodesmium* spp. blooms do not develop more often in the SEMS and the Mediterranean Sea (despite being routinely exposed to external organic and inorganic nutrients) remains unknown and merit further research.

## Conclusions

Our results provide the first recorded observation of a *Trichodesmium* (*T. erythraeum*) bloom in the coastal waters of the SEMS, which was stimulated by a prolonged and intense sewage outburst event. We suggest that the introduction of high amounts of N, P and DOC, as well as the C:N:P ratio measured here, triggered the formation of the *Trichodesmium* bloom. Specifically, assuming that *Trichodesmium* are mixotrophs, the high concentrations of DOC may have provided the energy sources to meet the metabolic requirements for N_2_ fixation and bloom formation. It should be noted that further and dedicated research is merit to test the metabolism strategies of *Trichodesmium*.

Following the above, our results indicate that anthropogenic contamination such as raw sewage outbursts into oligotrophic environments may not only trigger blooms of *T. erythraeum*, but also shift microbial community structure. It is likely that these sewage outburst events may have great ecological implications via *Trichodesmium* blooms in oligotrophic environments such as the SEMS. Yet, we stress that more controlled experiments and *in situ* monitoring are needed to better understand the dynamics, regulation and formation of *Trichodesmium* blooms in the SEMS.
